# Effect of Fluticasone propionate Aqueous Nasal Spray Treatment on Platelet Activating Factor and Eicosanoid Production By nasal Mucosa in Patients with A house Dust Mite Allergy

**DOI:** 10.1155/S0962935194000530

**Published:** 1994

**Authors:** I. M. Garrelds, T. de Graaf-in 't Veld, A. P. H. Jansen, R. Gerth van Wijk, F. J. Zijlstra

**Affiliations:** 1 Department of Pharmacology Faculty of Medicine Erasmus University Rotterdam The Netherlands; 2 Department of Allergology University Hospital Rotterdam-Dijkzigt P.O. Box 1738 Rotterdam 3000 DR The Netherlands

## Abstract

The relationship between the release of platelet activating factor (PAF), leukotriene C_4_/D_4_/E_E_ (LTC_4_/D_4_/E_4_) and prostaglandin D_2_ (PGD_2_) from nasal mucosa *in vivo* was examined in 24 rhinitis patients allergic to the house dust mite (HDM). During a double blind placebo controlled cross-over study 200 μg fluticasone propionate aqueous nasal spray (FPANS) was administered twice daily for two weeks. In response to allergen provocation (100, 1 000, 10 000 Bu/ml) and during the 9.5 h after this challenge the nasal fluid was obtained by washing the nose with saline and the levels of PAF, LTC_4_/D_4_/E_4_ and PGD_2_, as indicators of mediator release, were measured at the following time-points: baseline (t = − 1/2), allergen provocation with 10 000 Bu/ml (t = 0), 3.5 and 7.5 h (late phase). After allergen provocation the levels of the mediators increased in the nasal fluids of placebo treated patients (x-fold increase to baseline: PAF, 15; LTC_4_/D_4_/E_4_, 12; PGD_2_, 1.5). In fluids of patients treated with FPANS these levels tended to decrease. At the time of provocation the levels of PAF, LTC_4_/D_4_/E_4_ and PGD_2_ showed a significant correlation. The results indicate that these mediators can be used as markers of allergic reactions against house dust mites and that fluticasone propionate aqueous nasal spray tended to reduce the release of mediators of inflammation correlated with beneficial effects on clinical symptoms in this type of allergic reactions.


Mediators of Inflammation 3, 381–385 (1994)

### Effect of fluticasone propionate aqueous nasal spray treatment on platelet activating factor and eicosanoid production by nasal mucosa in patients with a house dust mite allergy

**I. M. Garrelds,[1,2,CA] T. de Graaf-in 't Veld,[2] A. P. H. Jansen,[2] R. Gerth van Wijk,[2] and F.J. Zijlstra[1]**

[1]Department of Pharmacology, Faculty of Medicine, Erasmus University Rotterdam; and [2]Department of Allergology, University Hospital Rotterdam-Dijkzigt, P.O. Box 1738, 3000 DR Rotterdam, The Netherlands

[CA] Corresponding Author

**THE relationship between the release of platelet activating factor (PAF), leukotriene $C_4/D_4/E_E$ ($LTC_4/D_4/E_4$) and prostaglandin $D_2$ ($PGD_2$) from nasal mucosa *in vivo* was examined in 24 rhinitis patients allergic to the house dust mite (HDM). During a double blind placebo controlled cross-over study 200 μg fluticasone propionate aqueous nasal spray (FPANS) was administered twice daily for two weeks. In response to allergen provocation (100, 1 000, 10 000 Bu/ml) and during the 9.5 h after this challenge the nasal fluid was obtained by washing the nose with saline and the levels of PAF, $LTC_4/D_4/E_4$ and $PGD_2$, as indicators of mediator release, were measured at the following time-points: baseline (t = – ½), allergen provocation with 10 000 Bu/ml (t = 0), 3.5 and 7.5 h (late phase). After allergen provocation the levels of the mediators increased in the nasal fluids of placebo treated patients (x-fold increase to baseline: PAF, 15; $LTC_4/D_4/E_4$, 12; $PGD_2$, 1.5). In fluids of patients treated with FPANS these levels tended to decrease. At the time of provocation the levels of PAF, $LTC_4/D_4/E_4$ and $PGD_2$ showed a significant correlation. The results indicate that these mediators can be used as markers of allergic reactions against house dust mites and that fluticasone propionate aqueous nasal spray tended to reduce the release of mediators of inflammation correlated with beneficial effects on clinical symptoms in this type of allergic reactions.**

**Key words**: Eicosanoids, Fluticasone propionate aqueous nasal spray, House dust mite, Platelet activating factor

### Introduction

In the 1920s house dust allergy was recognized when dust extracts from mattresses and vacuum cleaners were found to give relevant positive reactions in skin test on asthmatics.[1,2] Since 1964 it has been known that the majority of house dust sensitive patients show positive skin reactions to the mites of the genus *Dermatophagoides farinae* (Df) and *D. pteronyssinus* (Dp) as a major source in house dust.[3] The faeces particles, in particular, contain allergenic material in a concentrated form. Practicable control measures, such as chemicals, cleaning, ventilation and temperature regulation have only been able to reduce the number of mites in houses to some extent but the clinical effect has been disappointing.[1,3–6] As long as these methods are insufficient other forms of therapy are needed, such as immuno-therapy and symptomatic medication.[7–10]

House dust mites are the major cause of perennial rhinitis. The pathophysiology of allergic rhinitis, however, has been mainly studied in pollen allergy. Naclerio *et al.*[11] developed a model to explore the role of inflammatory mediators in ragweed pollinosis. As a consequence of cross-linking of IgE on mast cells and basophils by antigen mediators, such as prostaglandin $D_2$ ($PGD_2$), tryptase and histamine are released in the so-called early phase of the allergic process. These meditors cause sneezing, rhinorrhea and nasal congestion, which are the main symptoms of allergic rhinitis when they interact with neural elements, mucosal gland and blood vessels. After a quiescent period a second phase of the allergic process occurs. In this so-called late phase mediators are released again and symptoms recur.[11–14] The effect of systemic steroids, such as prednisone, reduce symptoms and mediator release in the late phase of the process. They have little or no effect on the early phase.[13,15] In contrast, topical steroids, such as flunisolide, used in the nose reduce symptoms and mediator release in the early phase as well in the late phase of the allergic process in a study with patients challenged with pollen antigens.[13,16] The corticosteroid, fluticasone propionate (FP) has potent topical anti-inflammatory activity coupled with low systemic activity. It has more than nine times the anti-inflammatory activity of fluocinolone acetonide and twice the activity of beclomethasone dipropionate.[17,18]

Nasal challenges and lavages were performed at the Department of Allergology and the measurements of platelet activating factor and eicosanoids at the Department of Pharmacology

© 1994 Rapid Communications of Oxford Ltd

The present study uses a nasal challenge model developed by Naclerio *et al.*[11] to explore the role of PAF and eicosanoids in the early and late phase of the allergic process in patients with allergic rhinitis against house dust mites. PAF could be involved in respiratory allergies because PAF is a potent eosinophil chemotactic factor.[19] However, to the authors' knowledge,, there are so far no available data regarding *in vivo* PAF generation by human nasal mucosa of patients allergic to house dust mites. In this report, the effect of fluticasone propionate aqueous nasal spray, a new and potent corticosteroid, on the levels of platelet activating factor (PAF), leukotriene $C_4/D_4/E_4$ ($LTC_4/D_4/E_4$) and prostaglandin $D_2$ ($PGD_2$) after nasal challenge with house dust mite extract, is also described.

### Materials and Methods

*Patients*: This study was performed in 24 patients. There were 11 women and 13 men aged 21 to 50 years (mean, 34 years). All were characterized by a history of perennial rhinitis, and by a positive skin test to house dust mite extract. All patients showed a skin reaction rated as at least one '+' sign to 0.3 to 3 Bu/ml extract, according to the standardized plus-sign scoring system defined by Norman.[20] Six of the 24 patients were allergic to grass pollen or animal dander as well. The nasal lavage experiments were performed between January and August to minimize exposure to house dust mites. The only patient with a concomitant pollen allergy was tested outside the pollen season. None of the patients allergic to animals had pets in their home. Antihistamines were withdrawn 72 h before testing. The antihistamine astemizole, topical corticosteroids, cromoglycate or nedocromil were not used for 3 weeks before the tests were performed. Oral corticosteroids had to be withdrawn 2 months before the study. Patients who developed a nasal infection during the 2-week period before entering the study were excluded. None had undergone immunotherapy previously.

The study was approved by the Medical Ethical Committee of the University Hospital Rotterdam-Dijkzigt and all patients gave written informed consent.

*Nasal challenge and lavage:* After the positive skin test the subjects entered the double blind placebo controlled crossover phase of the study. Each underwent two allergen challenges, performed after 2 weeks pretreatment with 200 µg fluticasone propionate aqueous nasal spray (FPANS) (Glaxo, GRD) or placebo spray twice daily. A 3-week washout period separated the two challenges. Before nasal challenge with house dust mite extract a nasal lavage was performed four times to obtain baseline mediator levels and to clear the nose from secretions. To prevent nasal congestion caused by the allergen challenges 0.250 ml oxymetazoline (0.1%) was sprayed into each nostril 5 min before the first challenge. Nasal lavage was performed as described by Naclerio *et al.* and Gerth van Wijk *et al.*[11,21,22] Both nostrils were washed with 5 ml saline, prewarmed to 37°C. Lavage fluid was collected in plastic tubes that were kept on ice. These lavage fluids were centrifuged for 10 min at 400 × **g** and the supernatants were stored at –70°C until detection of PAF or eicosanoids. To obtain a control challenge, 0.125 ml phosphate buffered saline (PBS) was sprayed in each nostril and a nasal lavage was performed. For allergen challenge 0.125 ml allergen extract was sprayed in each nostril and after 10 min thereafter a nasal lavage was performed. Allergen doses of 100, 1 000, 10 000 Biological Units (Bu)/ml (extract of *Dermatophagoides pteronyssinus*; ALK, Groningen, The Netherlands) were administered. From 30 min up to 9.5 h after this challenge the nasal fluid was obtained every hour by washing the nose with saline. Allergen-induced secretion collected before nasal lavage was not used for analysis. From the series of lavages the levels of PAF, $LTC_4/D_4/E_4$ and $PGD_2$, as indicators of mediator release, were measured at the following time points: baseline ($t = -½$), allergen provocation with 10 000 Bu/ml ($t = 0$), 3.5 and 7.5 h. These time points were chosen based on recently described studies,[21,22] in which it was shown that between 3 and 10 h after antigen challenge the late phase reaction occurred.

*Symptom score:* Symptoms were scored according to a scoring system described by Lebel *et al.*[23] These symptoms were observed in order to study the correlation between these clinical symptoms and the inflammatory mediators. The score was compiled before each lavage and after PBS and each allergen insufflation.

*Mediator assays:* The levels of PAF, $LTC_4/D_4/E_4$ and $PGD_2$ were measured by Scintillation Proximity Assay (SPA), Biotrak$^R$ and Radioimmunoassay (RIA) respectively (Amersham, UK). The limits of sensitivity of the assays were approximately 20, 3.1 and 0.75 pg/100 µl respectively. Cross-reactivity (50% $B/B_0$ displacement) of: PAF assay, 1-hexadecyl-2-acetyl GPC-PAF(C16:0) (100%), 1-octadecyl-2-acetyl GPC-PAF(C18:0) (40%), racPAF (29%), 1-hexadecyl-2-lyso GPC–Lyso–PAF(C16:0) (< 0.01%); $LTC_4/D_4/E_4$ assay: $LTC_4$ (100%), $LTD_4$ (100%), $LTE_4$ (70%), $LTB_4$ (0.4%) and prostaglandins (< 0.006%); $PGD_2$ assay, $PGD_2$ (100%), $PGJ_2$ (7%), $TxB_2$ (0.3%), $PGF_{2\alpha}$ (0.04%) and other prostaglandins (< 0.02%).

*Statistical analysis:* Statistical analysis was performed with the Friedman two-way ANOVA followed by the Wilcoxon matched-pairs signed-ranks test. The Kruskal-Wallis rank test was used for correlations.

For testing equality of the carry-over effect, within-patient totals over the two treatment periods are used. There is said to be no significant carry-over effect if the means of these within-patient totals do not significantly differ between the two treatment-order groups. For this test a $p$-value < 10% is considered significant.

### Results

*Nasal mediator release:* The levels of the inflammatory mediators, PAF, $LTC_4/D_4/E_4$, and $PGD_2$, in nasal washings from allergic patients to house dust mites with and without fluticasone propionate aqueous nasal spray (FPANS) are presented in Table 1. No significant carry-over effect was observed. The baseline levels of the placebo group and FPANS group respectively are: PAF, 907 ± 177 (range 147–3172) and 780 ± 316 (range 95–7272) pg/ml; $LTC_4/D_4/E_4$, 112 ± 10 (range 37–233) and 106 ± 9 (range 10–209) pg/ml and $PGD_2$, 94 ± 26 (range 21–592) and 92 ± 30 (range 3–734) pg/ml. Because these baseline levels are in a large range, the levels are recalculated in percent change to baseline.

Nasal challenge with house dust mite extract caused an immediate influx of these inflammatory mediators. After allergen provocation the levels of the mediators increased in the nasal fluids of placebo treated patients (x-fold increase to baseline: PAF, 15; $LTC_4/D_4/E_4$: 12; and $PGD_2$, 1.5). In fluids of patients treated with FPANS these levels tended to decrease (x-fold increase to baseline: PAF, 6; $LTC_4/D_4/E_4$, 4; and $PGD_2$, 1.1). $p$ Value between the placebo and FPANS group after the primary trigger initiated after challenge with 10 000 Bu/ml house dust mite extract of PAF, 0.2124; $LTC_4/D_4/E_4$, 0.1618; and $PGD_2$, 0.2227. At 3.5 and 7.5 h after this challenge a significant decrease of PAF, $LTC_4/D_4/E_4$ and $PGD_2$ was observed in both groups compared with the value at the time point of allergen provocation with 10 000 Bu/ml house dust mite extract. The $p$ values of the placebo group and the FPANS group at 3.5 h are respectively: PAF, 0.0004 and 0.0010; $LTC_4/LTD_4/LTE_4$, 0.0003 and 0.0003; $PGD_2$, 0.0126 and 0.0116. At 7.5 h the $p$ values of the placebo group and the FPANS group are respectively: PAF, 0.0116 and 0.0184; $LTC_4/D_4/E_4$: 0.0300 and 0.0025; $PGD_2$, 0.0023 and 0.0936.

*Symptom score:* The means (±S.E.M.) of the symptom score are shown in Fig. 1. Because a significant carry-over effect was observed, only the results of the first treatment period was used. A significant increase is observed immediately after the challenge with house dust mite extract in the placebo and FPANS group. At 3.5 and 7.5 h after this challenge a significant decrease of the symptom score is observed as compared to the level at the time point of the challenge in both groups ($p$ = 0.001). The symptom score of patients treated with FPANS is decreased in comparison to the placebo group.

*Correlation between inflammatory mediators and symptom score:* A significant correlation ($p = \leq 0.05$) is found immediately after the challenge with 10 000

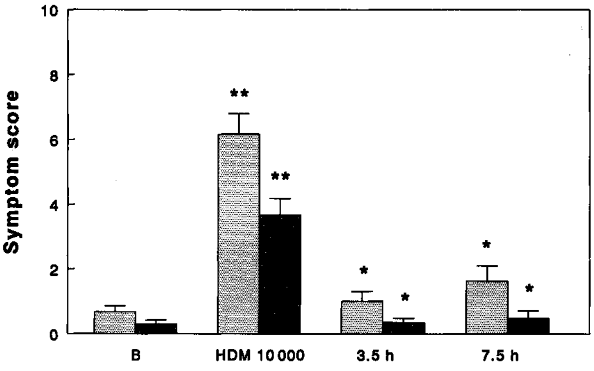

FIG. 1. Symptom score at the following time points: baseline (B), allergen provocation with 10 000 Bu/ml house dust mite extract (t = 0), 3.5 and 7.5 h (late phase) of allergic patients treated with or without FPANS. Values are expressed mean ± S.E.M. Statistical significant difference to the HDM 10 000 is shown by an asterisk. ($p \leq 0.05$). Statistical significant difference to the baseline is shown by a double asterisk ($p \leq 0.05$). Key: ▒, placebo; ▒, fluticasone propionate aqueous nasal spray.

**Table 1.** Platelet activating factor (PAF), leukotriene $C_4/D_4/E_4$ ($LTC_4/D_4/E_4$) and prostaglandin $D_2$ ($PGD_2$) production in nasal lavages

| | HDM 10 000 Bu/ml | | 3.5 h | | 7.5 h | |
|---|---|---|---|---|---|---|
| | Placebo | FPANS | Placebo | FPANS | Placebo | FPANS |
| PAF | 1490 ± 479 | 554 ± 283 | 5 ± 19* | 29 ± 37* | 19 ± 20* | 1 ± 28* |
| $LTC_4/D_4/E_4$ | 1115 ± 518 | 355 ± 190 | −4 ± 9* | −21 ± 7* | −6 ± 7* | −9 ± 9* |
| $PGD_2$ | 50 ± 26 | 8 ± 16 | −42 ± 11* | −42 ± 10* | −39 ± 8* | −29 ± 12 |

Results were obtained at the following time points: allergen provocation with 10 000 Bu/ml house dust mite (HDM) extract (t = 0), 3.5 and 7.5 h (late phase) of allergic patients treated with placebo or with fluticasone propionate aqueous spray (FPANS). Because of scattered individual data values are expressed as percent change of baseline ± S.E.M. Statistical significant decrease to the HDM 10 000 is shown with an asterisk, according to the Wilcoxon matched-pairs signed-ranks test ($p \leq 0.05$).

Bu/ml house dust mite extract between: (1) the release of $LTC_4/D_4/E_4$ and $PGD_2$ in the placebo group (correlation coefficient, $c = 0.656$) and in the FPANS group ($c = 0.776$); (2) the release of PAF and $LTC_4/D_4/E_4$ in the placebo group ($c = 0.719$) and in the FPANS group ($c = 0.990$); (3) the release of PAF and $PGD_2$ after the administration of placebo ($c = 0.466$) or FPANS ($c = 0.740$); (4) the release of $LTC_4/D_4/E_4$ and symptom score in the placebo group ($c = 0.547$) and in the FPANS group ($c = 0.545$); and (5) the release of PAF and symptom score of the FPANS group ($c = 0.598$).

### Discussion

Lavage of the nasal mucosa appears to be a convenient model for measuring inflammatory mediator release during an allergic reaction to the house dust mite. In agreement with other investigators it was found that within a few minutes of exposure to an allergen leukotrienes and prostaglandins can be measured in nasal washings.[11,24–28] This is the first study in which PAF could be measured in nasal lavages in detectable amounts seen within a few minutes after nasal provocation with house dust mite extract. Other investigators found lyso-PAF but almost no PAF by bioassay in nasal washings after nasal challenge of patients with a pollen allergy.[29,30]

*In vitro* studies have demonstrated that PAF is released by alveolar macrophages,[31] eosinophils,[32] monocytes and endothelial cells[34,35] and platelets.[36] It has now been demonstrated that PAF is present in nasal lavages of patients with house dust mite allergy; however, the origin of PAF is uncertain. In the early phase of the allergic process IgE crosslinks by antigen challenge on mast cells and basophils, which release primary mediators. After a quiescent period a late phase allergic reaction occurs, in which eosinophils and macrophages are involved, releasing secondary mediators.[11–14] It has been shown that PAF is released during the early phase reaction as primary mediator and not as a secondary mediator. The present study also shows that $PGD_2$ is released only during the early phase of the allergic process as a primary mediator. This is in agreement with other investigators, who found that $PGD_2$ is produced by mastcells.[37,38] Sulfidopeptide-leukotrienes are known to be released by eosinophils[39–41] and macrophages,[42–44] which indicated that $LTC_4/LTD_4/LTE_4$ are secondary mediators. However, these sulfidopeptide-leukotrienes were released only during the early phase reaction and not during the late phase reaction, which indicated that these sulfidopeptide-leukotrienes are also primary mediators. The generation of the mediators PAF, $LTC_4/D_4/E_4$ and $PGD_2$, reached baseline levels after 3.5 h. During the late phase reaction symptoms partially recurred, but surprisingly PAF, $LTC_4/D_4/E_4$ and $PGD_2$ were not released again. These symptoms should be due to other mediators. Furthermore it was clearly demonstrated that the release of PAF, $LTC_4/D_4/E_4$ and $PGD_2$ correlated with each other immediately at the time point of the nasal challenge with house dust mite extract. Previous reports of studies with patients allergic to grass pollen and ragweed described a correlation between $LTC_4/D_4/E_4$ and $PGD_2$.[23,45] We also found a significant correlation between the release of $LTC_4/D_4/E_4$ and PAF with the symptom score at the time point of challenge, but not between the release of $PGD_2$ and the symptom score. However, Lebel *et al.*[23] who studied patients with a grass pollen allergy observed that the release of $PGD_2$ was well correlated with the symptom score.

In this study pretreatment of the patients with FPANS for 2 weeks twice daily greatly reduced the development of symptoms. In a study performed with 17 atopic patients, during a 2 week pretreatment with FPANS 200 μg/day the immediate increase in nasal airway resistance was not inhibited.[46] In another study the dose of ragweed pollen required to produce a standardised response was unchanged after 4 weeks of treatment with FPANS 200 μg/day in 49 patients during the ragweed season.[47] However, FPANS improved the symptom score after 2 and 4 weeks of treatment in 24 patients with seasonal allergic rhinitis after nasal challenge with allergen.[48]

It has been suggested that the number of eosinophils and basophilic cells (basophils and mast cells) increase following allergen challenge and that this factor is responsible for the initiation of the allergic vascular response.[49,50] Treatment with FPANS 50 to 800 μg/day administered for 2 weeks to 6 months was associated with a significant decrease in the number of nasal eosinophils, basophils and neutrophils compared with placebo patients with seasonal allergic rhinitis.[51] It has been proposed that FPANS may act by preventing activation of several cells and subsequent release of inflammatory mediators.[30]

The present findings indicate that FPANS reduces not only the allergen induced symptoms (ratio placebo:FPANS, 1.68) but also tended to reduce the release of PAF (ratio placebo:FPANS, 2.69), $LTC_4/D_4/E_4$ (ratio placebo:FPANS, 3.14) and $PGD_2$ (ratio placebo:FPANS, 6.25) after the primary trigger initiated after challenge with 10 000 Bu/ml house dust mite extract at the time point of challenge.

In conclusion, the results indicate that the inflammatory mediators platelet activating factor, leukotriene $C_4/D_4/E_4$ and prostaglandin $D_2$ can be used as markers of allergic reactions to house dust mites and that fluticasone propionate aqueous nasal spray counteracts the release of mediators of inflammation, correlated with beneficial effects on clinical symptoms in this type of allergic reaction.

### References

1. Mosbech M. House dust mite allergy. *Allergy* 1985; **40**: 81–91.
2. Kern RA. Dust sensitization in bronchial asthma. *Med Clin North Am* 1921; **5**: 751–758.
3. Voorhorst R, Spieksma-Boezeman MIA, Spieksma FThM. Is a mite (Dermatophagoides sp.) the producer of the house-dust allergen? *Allergie Asthma* 1964; **10**: 329–334.
4. Larson DG, Mitchell WF, Wharton GW. Preliminary studies on *Dermatophagoides farinae* Hughes, 1961 (Acari and house dust allergy). *J Med Entomol* 1969; **6**: 295–299.
5. Voorhorst R, Spieksma FThM, Varekamp H, Leupen MJ, Lyklema AW. The house dust mite (*Dermatophagoides pteronyssinus*) and the allergen it produces. Identify with the house-dust allergen. *J Allergy* 1967; **39**: 325–339.
6. Heinig JH, Mosbech H, Haugaard L. Diagnosis of house dust mite allergy. *Allergy* 1991; **46(Suppl 11)**: 19–22.
7. British Tuberculosis Association (Research Committee). Treatment of house dust allergy. *Br Med J* 1968; **3**: 774–777.
8. Gaddie J, Skinner C, Palmere KNV. Hyposensitisation with house dust mite vaccine in bronchial asthma. *Br Med J* 1976; **2**: 561–562.
9. Formgren H, Lanner A,. Lindholm N, Lovhagen O, Dreborg S. Bronchial and nasal sensitivity changes during one year of immunotherapy with mite extracts. *Folia Allergol Immunol Clin* 1983; XXX: Suppl: al N4.
10. Gabriel SMNgHK, Allan WGL, Hill LE, Nunn AJ. Study of prolonged hyposensitization with *D. pteronyssinus* extract in allergic rhinitis. *Clin Allergy* 1977; **7**: 325–336.
11. Naclerio RM, Meier HL, Kagey-Sobatka A, *et al.* Mediator release after nasal airway challenge with allergen. *Am Rev Respir Dis* 1983; **128**: 597–602.
12. Naclerio RM, Proud D, Peters SP, *et al.* Inflammatory mediators in nasal secretions during induced rhinitis. *Clin Allergy* 1986; **16**: 101–110.
13. Naclerio RM. The pathophysiology of allergic rhinitis: Impact of therapeutic intervention. *J Allergy Clin Immunol* 1988; **82**: 927–934.
14. Van Toorenenbergen AW, Gerth van Wijk R, Vermeulen AM, Zijlstra FJ. Increase of albumin, eosinophil cationic protein, histamine, leukotrienes and mast cell tryptase in nasal lavage fluids after challenge with inhalant allergen extract. *Agents Actions* 1992; Special Conference Issue: C421–C424.
15. Pipkorn U, Proud D, Lichtenstein LM, *et al.* Effect of short-term systemic glucocorticoid treatment on human nasal mediator release after antigen challenge. *J Clin Invest* 1987; **80**: 957–961.
16. Pipkorn U, Proud D, Lichtenstein LM, *et al.* Topical steroid pretreatment inhibits mediator release *in vivo*. *J Engl J Med* 1987; **316**: 1506–1510.
17. Phillipps GH. Structure–activity relationships of topical active steroids; the selection of fluticasone propionate. *Respir Med* 1990; **84(Suppl A)**: 19–23.
18. Bryson HM, Faulds, D. Intranasal fluticasone propionate; a review of its pharmacodynamic and pharmacokinetic properties and therapeutic potential in allergic rhinitis. *Drugs* 1992; **43**: 760–775.
19. Wardlaw AJ, Moqbel R, Cromwell O, Kay AB. Platelet-activating factor: a potent chemotactic and chemokinetic factor for human eosinophils. *J Clin Invest* 1986; **78**: 1701–1706.
20. Norman PS. Skin testing. In: Rose NR, Friedman H eds. *Manual of clinical immunology. 2nd edn.* Washington: American Society for Microbiology, 1980: 789–793.
21. Gerth van Wijk R, Zijlstra FJ, Van Toorenenbergen AW, Vermeulen A, Dieges PH. Isolated early response after nasal allergen challenge is sufficient to induce nasal hyperreactivity. *Ann Allergy* 1992; **69**: 43–47.
22. Gerth van Wijk R, Van Toorenenbergen AW, Zijlstra FJ, Jansen APH, Dieges PH. Nasal hyperreactivity and its effects on early and late sequela of nasal challenge with house dust mite extract. *Allergy Proc* 1993; **14**: 273–281.
23. Lebel B, Bousquet J, Morel A, *et al.* Correlation between symptoms and the threshold for release of mediators in nasal secretions during challenge with grass-pollen grains. *J Allergy Clin Immunol* 1988; **82**: 869–877.
24. White MV, Kaliner MA. Mediators of allergic rhinitis. *J Allergy Clin Immunol* 1992; **90**: 699–704.
25. Bisgaard H, Robinson C, Rømeling F, Mygind N, Church M, Holgate ST. Leukotriene $C_4$ and histamine in early allergic reaction in the nose. *Allergy* 1988; **43**: 219–227.
26. Ophir D, Fink A, Eliraz A, Tabachnick E, Bentwich Z. Allergen-induced leukotriene production by nasal mucosa and peripheral blood leukocytes. *Arch Otolaryngol Head Neck Surg* 1988; **114**: 522–524.
27. Brown MS, Peters SP, Adkinson F, *et al.* Arachidonic acid metabolites during nasal challenge. *Arch Otolaryngol Head Neck Surg* 1987; **113**: 179–183.
28. Norman PS, Naclerio RM, Creticos PS, Togias, Lichtenstein LM. Mediators release after allergic and physical nasal challenges. *Int Archs Allergy Appl Immun* 1985; **77**: 57–63.
29. Miadonna A, Tedeschi A, Arnoux B, Sala A, Zanussi C and Benveniste J. Evidence of PAF-acether metabolic pathway activation in antigen challenge of upper respiratory airways. *Am Rev Respir Dis* 1989; **140**: 142–147.
30. Meslier N, Braunstein G, Lacronique J, *et al.* Local cellular and humoral responses to antigenic and distilled water challenge in subjects with allergic rhinitis. *Am Rev Respir Dis* 1988; **137**: 617–624.
31. Arnoux B, Joseph M, Simoes MH, *et al.* Antigenic release of PAF-acether and glucuronidase from alveolar macrophages of asthmatics. *Bull Eur Physiopathol Respir* 1987; **23**: 119–124.
32. Lee TC, Lenihan DJ, Malone B, Roddy LL, Wasserman SI. Increased biosynthesis of platelet activating factor from activated human eosinophils. *J Biol Chem* 1984; **259**: 4436.
33. Lotner GZ, Lynch JM, Betz ST, Henson PM. Human neutrophil-derived platelet activating factor. *J Immunol* 1980; **124**: 676–684.
34. Camussi G, Bussolino F, Tetta C, Piacibello W, Anglietta M. Biosynthesis and release of platelet-activating factor from human monocytes. *Int Arch Allergy Appl Immunol* 1983; **70**: 245–251.
35. Camussi G, Anglietta M, Malavasi F, *et al.* The release of platelet-activating factor from human endothelial cells in culture. *J Immunol* 1983; **131**: 2397–2403.
36. Chignard M, Le Couedic JP, Vargaftig BB, Benveniste J. Platelet activating factor (PAF-acether) secretion from platelets. Effect of various aggregating agents. *Br J Haematol* 1980; **46**: 455–464.
37. Benyon RC, Robinson C, Church MK. Differential release of histamine and eicosanoids from human skin mast cells activated by IgE-dependent and non-immunological stimuli. *Br J Pharmacol* 1989; **97**: 889–904.
38. Wenzel SE, Fowler AA, Schwartz LB. Activation of pulmonary mast cells by bronchoalveolar lavage allergen challenge. *In vivo* release of histamine and tryptase in atopic subjects with and without asthma. *Am Rev Respir Dis* 1988; **137**: 1002–1008.
39. Weller PF, Lee CW, Foster DW, Corey EJ, Austen KF, Lewis RA. Generation and metabolism of 5-lipoxygenase pathway leukotrienes by human eosinophils: predominant production of leukotriene $C_4$. *Proc Natl Acad Sci USA* 1983; **80**: 7626–7630.
40. Shaw RJ, Cromwell O, Kay AB. Preferential generation of leukotriene $C_4$ by human eosinophils. *Clin Exp Immunol* 1984; **56**: 716–722.
41. Moqbel R, MacDonald AJ, Cromwell O, Kay AB. Release of leukotriene $C_4$ ($LTC_4$) from human eosinophils following adherence to IgE- and IgG-coated schistosomula of *Schistosoma mansoni*. *Immunology* 1990; **69**: 435–442.
42. Balter MS, Toews GB, Peters-Golden M. Different pattern of arachidonate metabolism in autologous human blood monocytes and alveolar macrophages. *J Immunol* 1989; **142**: 602–608.
43. Ouwendijk RJTh, Zijlstra FJ, Van den Broek AMWC, Brouwer A, Wilson JHP, Vincent JE. Comparison of the production of eicosanoids by human and rat peritoneal macrophages and rat Kupffer cells. *Prostaglandins* 1988; **35**: 437–446.
44. Bonney RJ, Humes JL. Physiological and pharmacological regulation of prostaglandin and leukotriene production by macrophages. *J Leukocyte Biol* 1984; **35**: 1–10.
45. Georgitis JW, Stone BD, Gottschlich G. Nasal inflammatory mediator release in ragweed allergic rhinitis: correlation with cellular influx into nasal secretions. *Int Archs Allergy Appl Immun* 1991; **96**: 231–237.
46. Thomas KE, Greenwood L, Murrant N, *et al.* The effect of topical fluticasone propionate on allergen-induced immediate nasal airways response and eosinophil activation: preliminary results. *Respir Med* 1990; **84(Suppl A)**: 33–35
47. Small P, Biskin N, Barrett D. The effects of intranasal fluticasone propionate on allergen induced nasal provocation. *Clin Invest Med* 1989; **12(Suppl 4)**: b5.
48. Holmberg K, Juliusson S, Karlsson G, *et al.* Effects of 4 weeks treatment with the topical glucocorticoid fluticasone on allergen-induced symptoms, tissue mast cells and histamine in the nasal mucosa. *XIV Int Congress of Allergol and Clin Immunol* 1991: 183.
49. Pipkorn Ul Karlsson G, Enerback L. The cellular response of the human allergic mucosa to natural allergen exposure. *J Allergy Clin Immunol* 1988; **82**: 1046–1054.
50. Howarth PH, Rajakulasingam K, Feather IH. Mediators and allergic rhinitis. *Clin Exp Allergy* 1991: **21:(Suppl 1)**: 262–266.
51. Meltzer EO, Orgel HA, Bronsky EA, *et al.* A dose-ranging study of fluticasone propionate aqueous nasal spray for seasonal allergic rhinitis assessed by symptoms, rhinomanometry, and nasal cytology. *J Allergy Clin Immunol* 1990; **86**: 221–230.

ACKNOWLEDGEMENTS: This study was supported by Glaxo B.V., The Netherlands. The advice and assistance of Paul G.M. Mulder PhD, of the Dept of Epidemiology and Biostatistics on statistical methods is gratefully acknowledged.

**Received 17 May 1994; accepted in revised form 17 June 1994**
